# Supplementary material for: SAMHD1 deacetylation by SIRT1 promotes DNA end resection by facilitating DNA binding at double-strand breaks
Source: Nat Commun. 2022 Nov 7;13:6707. doi: 10.1038/s41467-022-34578-x (PMC9640623; doi:10.1038/s41467-022-34578-x)
Supplement: Supplementary file 1 — Supplementary Information [file 41467_2022_34578_MOESM1_ESM.pdf]

Supplementary Information

SAMHD1 deacetylation by SIRT1 promotes DNA end resection by facilitating DNA binding at double-strand breaks

Priya Kapoor-Vazirani[1], Sandip K. Rath[1], Xu Liu[2], Zhen Shu[1], Nicole E. Bowen[3], Yitong Chen[1], Ramona Haji-Seyed-Javadi[1], Waaqo Daddacha[4], Elizabeth V. Minten[1], Diana Danelia[1], Daniela Farchi[1], Duc M. Duong[2], Nicholas T. Seyfried[2], Xingming Deng[1], Eric A. Ortlund[2], Baek Kim[3], and David S. Yu[1*]

[1] Department of Radiation Oncology and Winship Cancer Institute, Emory University School of Medicine, Atlanta, GA 30322, USA
[2] Department of Biochemistry, Emory University School of Medicine, Atlanta, GA 30322, USA
[3] Department of Pediatrics, Emory University School of Medicine, Atlanta, GA 30322, USA
[4] Department of Biochemistry and Molecular Biology, Medical College of Georgia at Augusta University, Augusta, GA 30912, USA

*Correspondence: dsyu@emory.edu

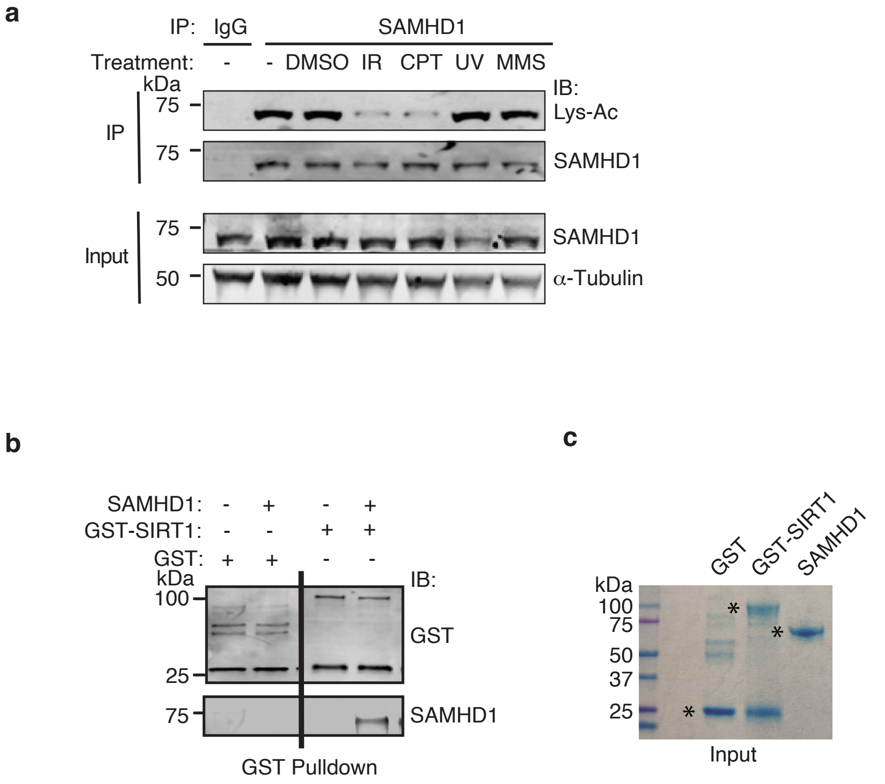

Figure S1. SAMHD1 is deacetylated in response to DSBs through direct interaction with SIRT1. **a** 293T cells were treated with the indicated agents and harvested for IP with either IgG (negative control) or anti-SAMHD1 antibody. IP'ed lysates were separated by SDS-PAGE and immunoblotted with the indicated antibodies. **b** Recombinant GST or GST-SIRT1 (aa 193-741) proteins were incubated with recombinant SAMHD1 at equimolar amounts and subjected to in-vitro GST pulldown with glutathione-agarose beads. Shown are western analysis of the GST pulldown lysates blotted with anti-GST or SAMHD1 antibodies. **c** Coomassie-stain of GST, GST-SIRT1, and SAMHD1 input used in b. Source data are provided as a Source Data file.

a

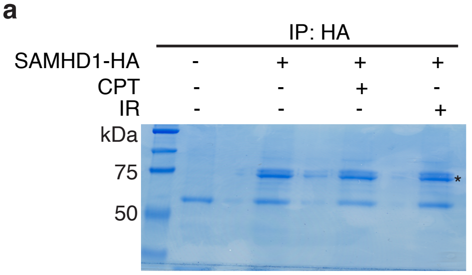

b

Modification	Peptide Sequence	Peptide Intensity			
		-SAMHD1-HA	+SAMHD1-HA		
		NT	NT	CPT	IR
K354ac	DKacEVGNLYDMFHTR	0	35622951	0	0
K494ac	LKacAEDFIVDVINMDYGMQEK	0	0	0	15416554
K580ac	FTKacPQDGDVIAPLITPQKK	0	0	0	8018827

Figure S2. SAMHD1 is deacetylated at K354 in response to DSBs. a Coomasie blue stained gel showing enrichment of SAMHD1-HA IP'ed from 293T cells treated with or without CPT or IR. Coomassie blue stained bands corresponding to SAMHD1-HA (marked by *) were excised for MS/MS. **b** Excised gel were subjected to MS/MS analysis to determine peptide intensity spectra. Intensity of peptides containing K354, K494 or K580 modified by acetylation is shown under treated and non-treated conditions.

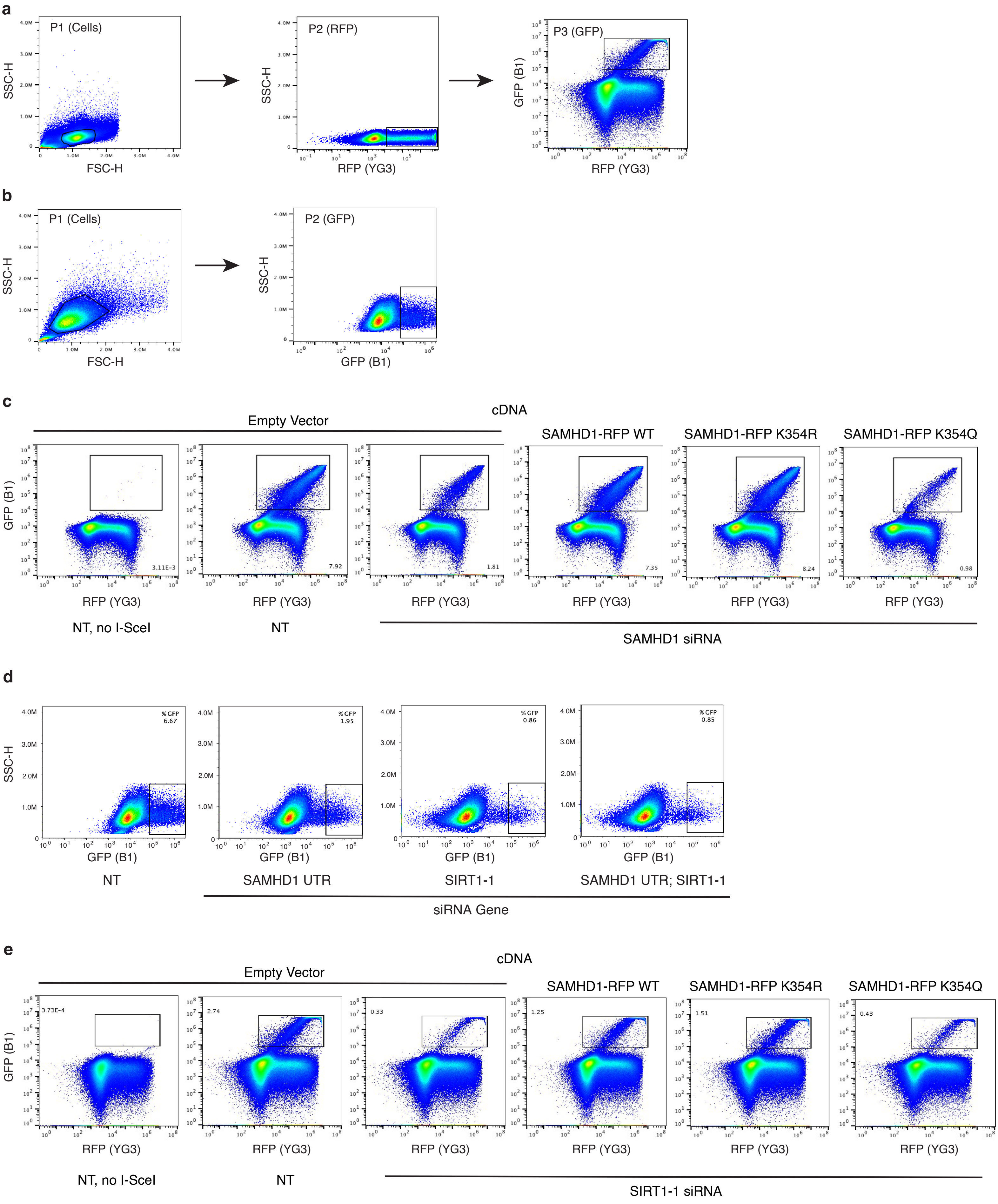
a
P1 (Cells)
SSC-H
FSC-H
P2 (RFP)
SSC-H
RFP (YG3)
P3 (GFP)
GFP (B1)
RFP (YG3)
b
P1 (Cells)
SSC-H
FSC-H
P2 (GFP)
SSC-H
GFP (B1)
c
cDNA
Empty Vector
SAMHD1-RFP WT
SAMHD1-RFP K354R
SAMHD1-RFP K354Q
GFP (B1)
3.11E-3
7.92
1.81
7.35
8.24
0.98
RFP (YG3)
RFP (YG3)
RFP (YG3)
RFP (YG3)
RFP (YG3)
RFP (YG3)
NT, no I-SceI
NT
SAMHD1 siRNA
d
SSC-H
% GFP
6.67
% GFP
1.95
% GFP
0.86
% GFP
0.85
GFP (B1)
GFP (B1)
GFP (B1)
GFP (B1)
NT
SAMHD1 UTR
SIRT1-1
SAMHD1 UTR; SIRT1-1
siRNA Gene
e
cDNA
Empty Vector
SAMHD1-RFP WT
SAMHD1-RFP K354R
SAMHD1-RFP K354Q
GFP (B1)
3.73E-4
2.74
0.33
1.25
1.51
0.43
RFP (YG3)
RFP (YG3)
RFP (YG3)
RFP (YG3)
RFP (YG3)
RFP (YG3)
NT, no I-SceI
NT
SIRT1-1 siRNA

Figure S3. SAMHD1 deacetylation at K354 by SIRT1 promotes HR. a. Sequential gating strategy used in the flow cytometry experiments conducted in c and e are shown. Samples were first gated for cells (P1) and within the cell population, cells expressing RFP (YG3 channel) were gated (P2). Then, within the RFP population, cells expressing GFP (B1 channel) were gated (P3). Plots of GFP vs. RFP (ie. P3 panel) showing RFP and GFP positive cells (boxed) are shown in c and e. **b.** Sequential gating strategy used in flow cytometry experiment conducted in d is shown. Samples were first gated for cells (P1) and then the cell population was gated for GFP expression (P2). P2 panel showing GFP positive cells (boxed) is represented in d. **c-e** Representative images of the flow cytometry analyses done in Figure 3b, g and i, respectively. U20S DR-GFP HR reporter cells were transfected with indicated siRNAs, cDNAs (where indicated) and I-SceI and subjected to flow cytometry. GFP positive cells (boxed) serve as an output for HR efficiency. SSC-H = side scatter; FSC-H = forward scatter.

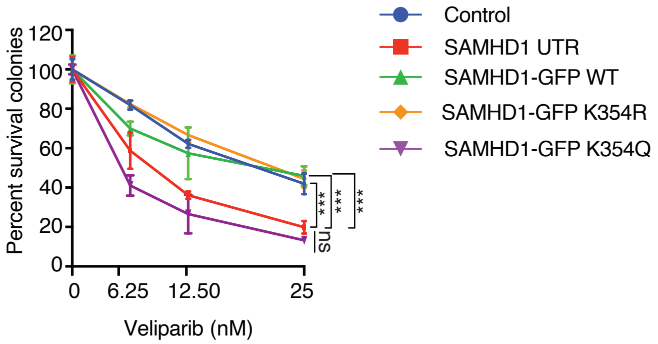

Figure S4. SAMHD1 deacetylation at K354 mediates PARP inhibitor resistance. U2OS cells silenced for endogenous SAMHD1 and expressing SAMHD1-GFP WT, K354R or K354Q were treated with indicated concentrations of veliparib and assayed for colony survival 10 -12 days later. The mean +/- SEM from 3 independent replicas is shown. P values (*** $p < 0.001$) were determined by two-way ANOVA followed by a Bonferroni test. Source data are provided as a Source Data file.

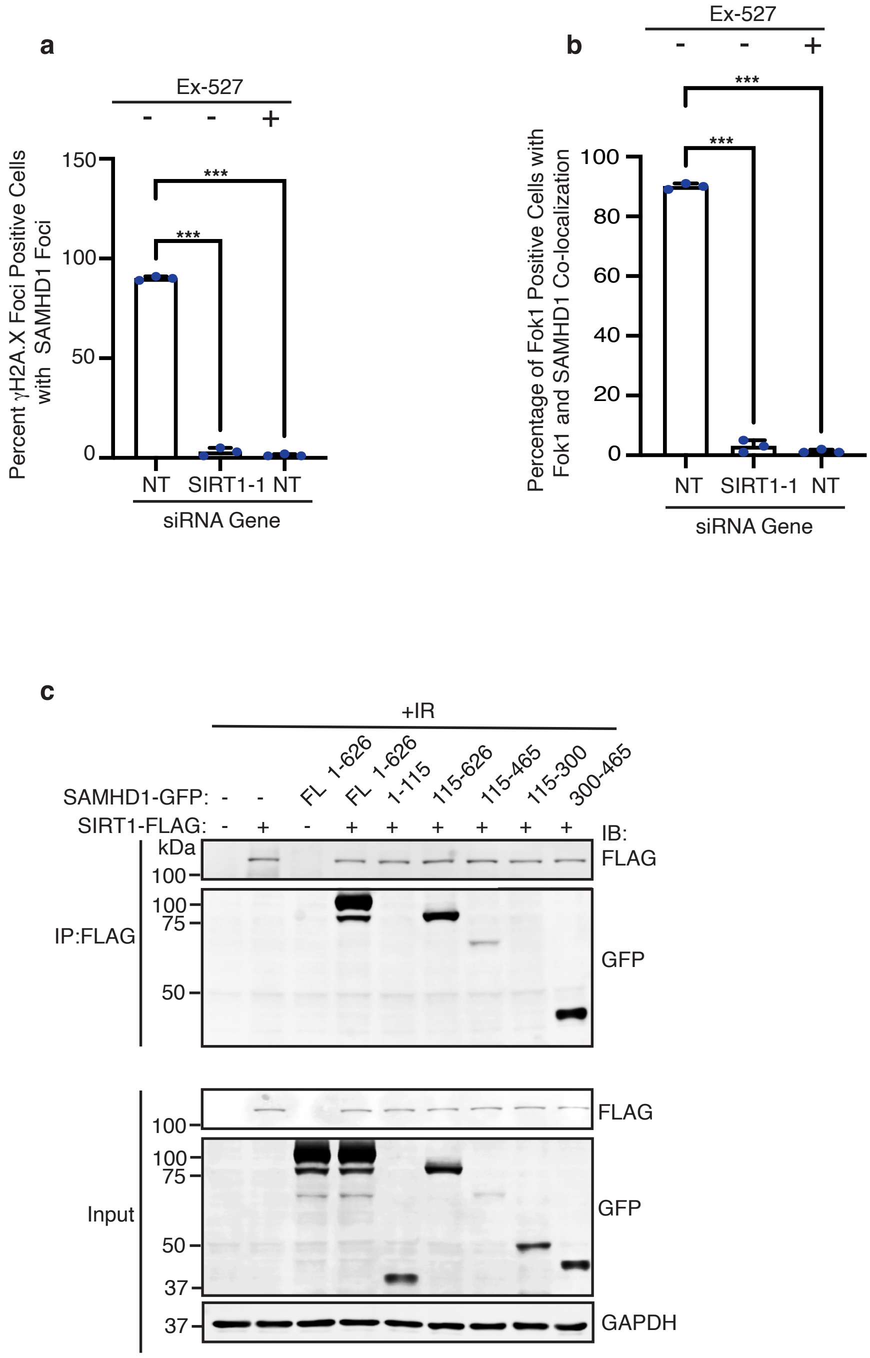

Figure S5. SIRT1 promotes SAMHD1 localization to DSBs. a-b Quantification of the representative images shown in Figure 5a (a) and 5b (b). Quantification is an average of three replicates, each containing 50 cell counts. Mean and SD are shown. P values (*** $p < 0.001$) were determined using Ordinary one-way ANOVA with Dunnett's post hoc test analysis. **c.** 293T cells were transfected with constructs expressing SIRT1-FLAG and/or SAMHD1-GFP fragments shown in Fig. 5c. Cells were treated with 10 Gy IR for 4 hrs and harvested. Protein lysates were prepared and IP'ed with anti-FLAG antibody. Input and IP'ed proteins were probed with antibodies against FLAG, GFP and GAPDH (loading control). Source data are provided as a Source Data file.

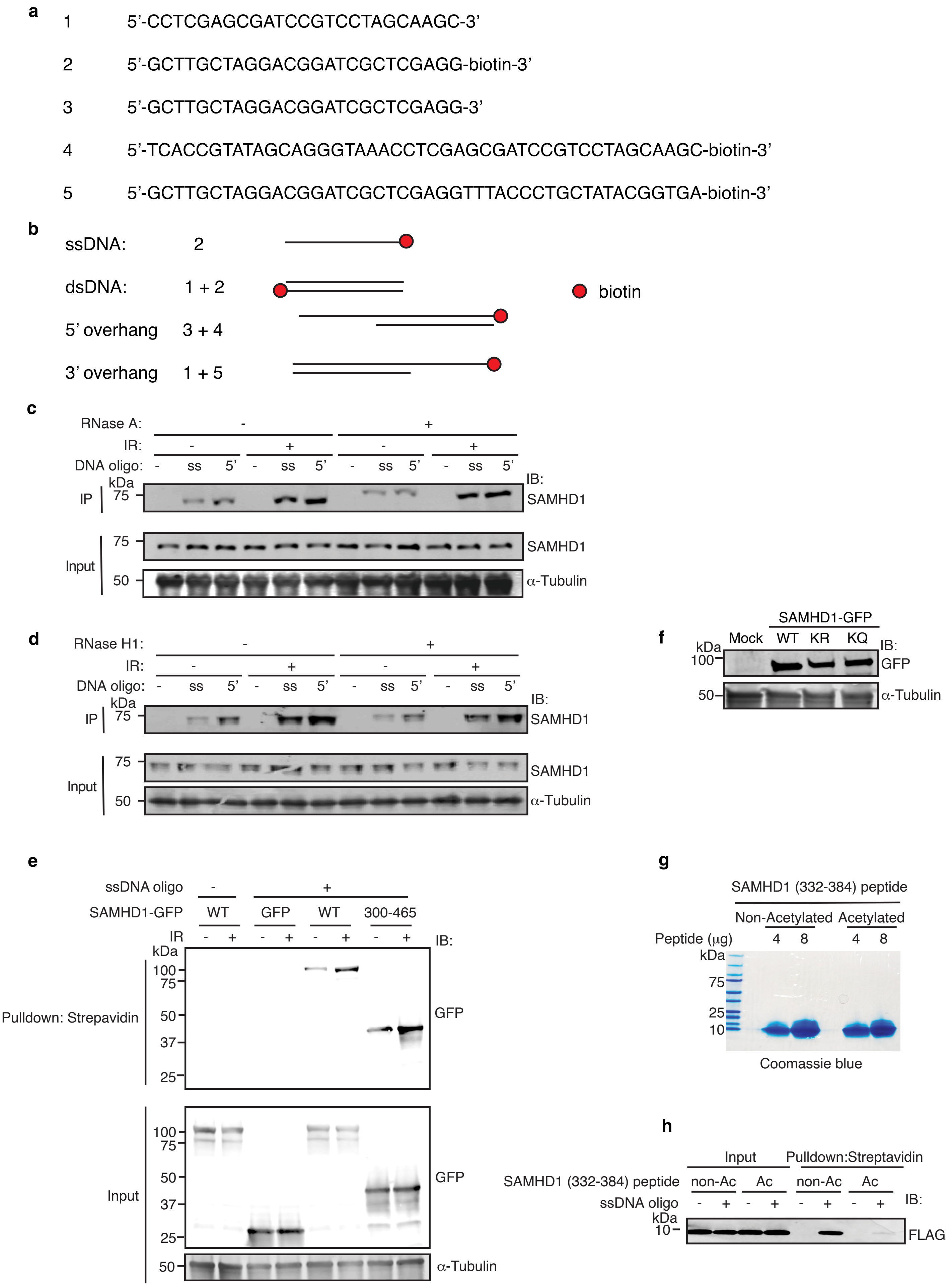

a
1 5'-CCTCGAGCGATCCGTCCTAGCAAGC-3'
2 5'-GCTTGCTAGGACGGATCGCTCGAGG-biotin-3'
3 5'-GCTTGCTAGGACGGATCGCTCGAGG-3'
4 5'-TCACCGTATAGCAGGGTAAACCTCGAGCGATCCGTCCTAGCAAGC-biotin-3'
5 5'-GCTTGCTAGGACGGATCGCTCGAGGTTTACCCTGCTATACGGTGA-biotin-3'
b
ssDNA: 2
dsDNA: 1 + 2
5' overhang 3 + 4
3' overhang 1 + 5
biotin
c
RNase A:
IR:
DNA oligo:
IP
Input
IB:
SAMHD1
α-Tubulin
d
RNase H1:
e
ssDNA oligo
SAMHD1-GFP
WT
GFP
300-465
Pulldown: Strepavidin
GFP
f
SAMHD1-GFP
Mock
WT
KR
KQ
g
SAMHD1 (332-384) peptide
Non-Acetylated
Acetylated
Peptide (μg)
Coomassie blue
h
Input
Pulldown:Streptavidin
non-Ac
Ac
FLAG

Figure S6. SAMHD1 binding to DNA is independent of RNA and RNA:DNA hybrids and is mediated by SAMHD1DNA damage recruitment domain 300-465. a Sequence of the oligonucleotides used to construct the DNA substrates used in Figure 6 are shown. Oligonucleotide sequences[1] are as previously described except that a biotin moiety was added to 3' end of some oligonucleotides as indicated. **b** Schematic of DNA substrates used in Figure 6 are indicated. Double-stranded regions (ds, 3' and 5' overhang substrates) are 25 bp long and overhangs (3' and 5' overhang substrates) are 20 bp long. ssDNA substrate is 25 bp long. **c-d** 293T cells and 293T cells subjected to 10 Gy IR for 4 hrs were treated with either RNase A or RNase H1, where indicated. Protein lysates were incubated with no DNA, ssDNA or 5' overhang DNA substrates and subjected to pull down with agarose-streptavidin beads. Proteins that bound DNA were separated on SDS-PAGE and western blotted for SAMHD1. Input protein lysates were also blotted with anti-SAMHD1 and α-Tubulin (control) antibodies. **e** 293T cell lysates expressing GFP, SAMHD1 WT or SAMHD1 300-465 and with or without IR were incubated with ssDNA oligo labeled with biotin. No oligo served as a control. Biotinylated ssDNA-protein complexes were pulled down and processed as described for c-d. GFP and α-tubulin antibodies were used for western analysis. **f** Western blot showing expression of SAMHD1-GFP WT, K354R, and K354Q in DIvA cells used in Figure 6h. **g** Coomassie blue staining of chemically synthesized FLAG-tagged SAMHD1 peptide 332-384 not acetylated or acetylated at K354. Expected size of the peptides is 7 kDa. **h** Non-acetylated (non-Ac) or acetylated (Ac) SAMHD1 peptides (8 μg) described in (g) was incubated with or without biotinylated ssDNA oligo. The mixture was pulled down with streptavidin-agarose beads, ran on SDS-PAGE and western blotted with anti-FLAG antibody to detect binding of the peptides to DNA oligo. Source data are provided as a Source Data file.

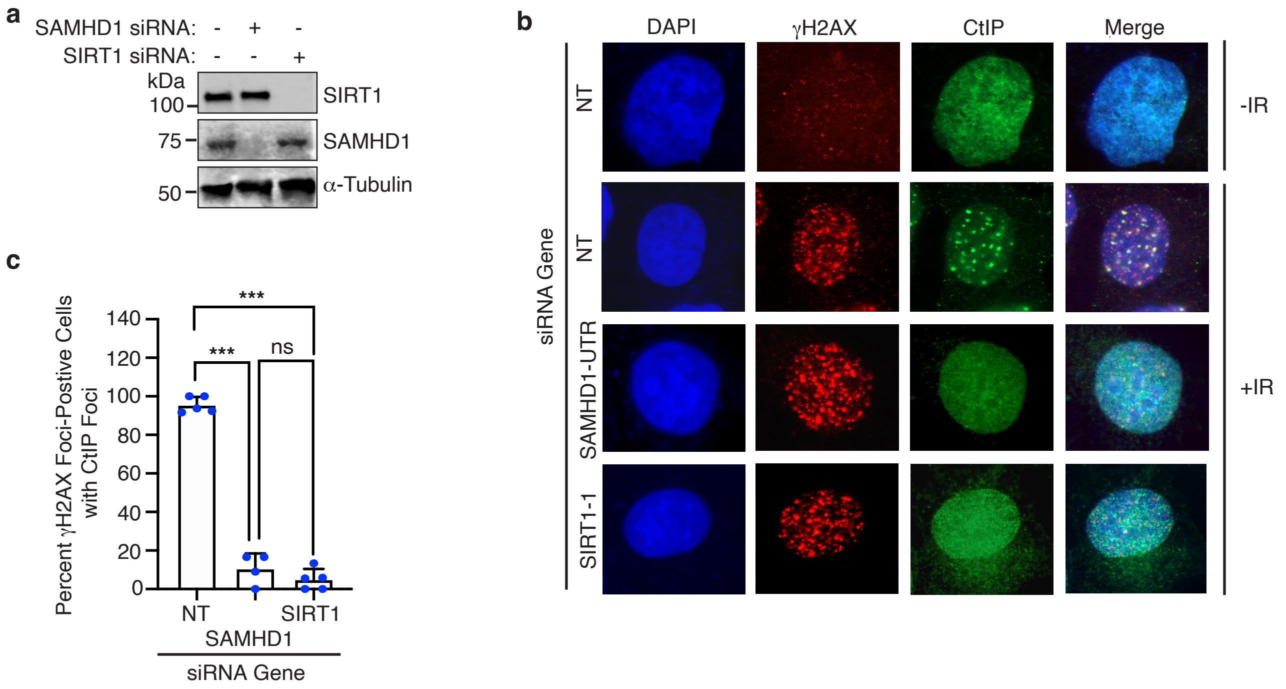

Figure S7. SIRT1 and SAMHD1 promote CtIP localization to DSBs. a Western blot showing downregulation of SAMHD1 and SIRT1 in cells used in b. **b** U2OS cells silenced for SAMHD1 or SIRT1 were subjected to immunofluorescence using antibodies against γH2AX and CtIP. DNA was stained with DAPI. Shown is a representative image. **c** γH2AX foci positive cells from b were scored for the presence or absence of CtIP foci. Shown is the mean and SD from 5 different fields of view (each field of view contained 5-16 cells). P values shown were determined using Ordinary one-way ANOVA with Dunnett's post hoc test analysis. *** $p < 0.001$; ns = non-significant. Source data are provided as a Source Data file.

Supplementary References

1 Wilkinson, O. J. *et al.* CtIP forms a tetrameric dumbbell-shaped particle which bridges complex DNA end structures for double-strand break repair. *Elife* **8**, doi:10.7554/eLife.42129 (2019).
