# Supplementary material for: SAMHD1 deacetylation by SIRT1 promotes DNA end resection by facilitating DNA binding at double-strand breaks
Source: Nat Commun. 2022 Nov 7;13:6707. doi: 10.1038/s41467-022-34578-x (PMC9640623; doi:10.1038/s41467-022-34578-x)
Supplement: Supplementary file 3 — Reporting Summary [file 41467_2022_34578_MOESM3_ESM.pdf]

nature portfolio

Corresponding author(s): Dr. David S. Yu

Last updated by author(s): Oct 12, 2022

Reporting Summary

Nature Portfolio wishes to improve the reproducibility of the work that we publish. This form provides structure for consistency and transparency in reporting. For further information on Nature Portfolio policies, see our Editorial Policies and the Editorial Policy Checklist.

Statistics

For all statistical analyses, confirm that the following items are present in the figure legend, table legend, main text, or Methods section.

n/a	Confirmed	
☐	☒	The exact sample size (*n*) for each experimental group/condition, given as a discrete number and unit of measurement
☐	☒	A statement on whether measurements were taken from distinct samples or whether the same sample was measured repeatedly
☐	☒	The statistical test(s) used AND whether they are one- or two-sided<br>*Only common tests should be described solely by name; describe more complex techniques in the Methods section.*
☒	☐	A description of all covariates tested
☒	☐	A description of any assumptions or corrections, such as tests of normality and adjustment for multiple comparisons
☐	☒	A full description of the statistical parameters including central tendency (e.g. means) or other basic estimates (e.g. regression coefficient) AND variation (e.g. standard deviation) or associated estimates of uncertainty (e.g. confidence intervals)
☐	☒	For null hypothesis testing, the test statistic (e.g. *F*, *t*, *r*) with confidence intervals, effect sizes, degrees of freedom and *P* value noted<br>*Give P values as exact values whenever suitable.*
☒	☐	For Bayesian analysis, information on the choice of priors and Markov chain Monte Carlo settings
☒	☐	For hierarchical and complex designs, identification of the appropriate level for tests and full reporting of outcomes
☒	☐	Estimates of effect sizes (e.g. Cohen's *d*, Pearson's *r*), indicating how they were calculated

Our web collection on statistics for biologists contains articles on many of the points above.

Software and code

Policy information about availability of computer code

Data collection	Western - LiCor Odyssey System<br>Flow Cytometry - Aurora Cytek<br>Microscopy - Leica SP8<br>RT-PCR - PCR 7500 Fast Real-Time PCR System (Thermo Fisher)<br>Mass Spec - Orbitrap Fusion<br>BioRad PharosFX imager
Data analysis	Western - ImageStudio 5.2<br>Flow Cytometry - FlowJo 10.8.1 and SpectroFlo 2.2.0.4<br>Microscopy - LasX 3.5.2.18963<br>Statistical Analysis and Graphing - GraphPad Prism 9<br>Mass Spec - Proteome Discoverer 2.0<br>Image Lab Software version 5.1.2

For manuscripts utilizing custom algorithms or software that are central to the research but not yet described in published literature, software must be made available to editors and reviewers. We strongly encourage code deposition in a community repository (e.g. GitHub). See the Nature Portfolio guidelines for submitting code & software for further information.

Data

Policy information about availability of data

All manuscripts must include a data availability statement. This statement should provide the following information, where applicable:

- Accession codes, unique identifiers, or web links for publicly available datasets
- A description of any restrictions on data availability
- For clinical datasets or third party data, please ensure that the statement adheres to our policy

Source data are provided with this paper. The mass spectrometry proteomics data have been deposited to the ProteomeXchange Consortium via the PRIDE partner repository with the dataset identifier PXD031357. Reviewer account details - Username: reviewer_pxd031357@ebi.ac.uk; Password: 9IoiSJom.

Human research participants

Policy information about studies involving human research participants and Sex and Gender in Research.

Reporting on sex and gender	N/A
Population characteristics	N/A
Recruitment	N/A
Ethics oversight	N/A

Note that full information on the approval of the study protocol must also be provided in the manuscript.

Field-specific reporting

Please select the one below that is the best fit for your research. If you are not sure, read the appropriate sections before making your selection.

☒ Life sciences ☐ Behavioural & social sciences ☐ Ecological, evolutionary & environmental sciences

For a reference copy of the document with all sections, see nature.com/documents/nr-reporting-summary-flat.pdf

Life sciences study design

All studies must disclose on these points even when the disclosure is negative.

Sample size	Sample sizes were chosen based on prior published studies (Daddacha, W. et al. SAMHD1 Promotes DNA End Resection to Facilitate DNA Repair by Homologous Recombination. Cell reports 20, 1921-1935, doi:10.1016/j.celrep.2017.08.008 2017) and in consultation with bio statistical support.
Data exclusions	No data was excluded.
Replication	All experiments in the study were replicated at least twice (except mass spectrometry which was done once) with consistent results. In general, western analysis and microscopy studies were replicated 2-5 times, DRG assays were repeated 2-3 times, size exclusion chromatography (SEC) and dNTP quantification studies were replicated twice, and ChIP experiments were replicated twice and performed in biological duplicates. EMSA was replicated 5 times.
Randomization	Cells in culture were treated with specific conditions/treatments and thus was not random. Analysis was randomized and validated by multiple lab personnel. Since the study does not involve patient or animal work but involves specific treatments of cultured cells and their outcome, randomization is not full relevant to the study.
Blinding	Cells in culture needed to be treated for specific conditions so this could not be blinded; however, analysis was blinded/random and validated by multiple lab personnel.

Reporting for specific materials, systems and methods

We require information from authors about some types of materials, experimental systems and methods used in many studies. Here, indicate whether each material, system or method listed is relevant to your study. If you are not sure if a list item applies to your research, read the appropriate section before selecting a response.

Materials & experimental systems

n/a	Involved in the study
☐	☒ Antibodies
☐	☒ Eukaryotic cell lines
☒	☐ Palaeontology and archaeology
☒	☐ Animals and other organisms
☒	☐ Clinical data
☒	☐ Dual use research of concern

Methods

n/a	Involved in the study
☒	☐ ChIP-seq
☐	☒ Flow cytometry
☒	☐ MRI-based neuroimaging

Antibodies

Antibodies used

SAMHD1 (Origene # TA502024; 1:1000 for Western, 1 uL per 1 mg lysate for IP; and Abcam # ab177462; 1:5000 for Western, 1:100 for IF); IgG (Invitrogen # 10500C and Sigma # N103); Pan acetylated lysine (Cell Signaling Technology # 9441S; 1:1000 for Western, 1 uL per 1 mg lysate for IP); GFP (Santa Cruz Tech # SC996; 1:1000 for Western; and Abcam #ab290; 1:5000 for Western, 1 ug per 2 mg lysate for IP); SirT1 (Abcam # 32441; 1:5000 for Western, 2 uL per 1 mg lysate for IP); SirT2 (ThermoFisher; 1:1000 for Western); SirT6 (Abcam # ab62768; 1:2000 for Western); SirT7 (Abcam # 62748; 1:1000 for Western); FLAG (Cell Signaling Technology # 2368S; 1:1000 for Western; and Santa Cruz Tech # sc51590; 1:1000 for Western); a-Tubulin (Sigma # T6074; 1:10,000 for Western); GAPDH (Sigma, G9545; 1:2000 for Western); SAMHD1 K354Ac (Pierce, custom made; 1:1000 for Western); gH2AX (Cell Signaling Technology # s139; 1:200 for IF; and Millipore # 05-636; 1:4000 for IF, 1:1000 for Western); BrdU (BD Biosciences # 347580; 1:200 for IF); RPA32 (Santa Cruz Tech # sc-14692; 1:400 for Western); pRPA32 S4/8 (Bethyl # A700-009; 1:1000 for Western); CtIP (Millipore # MABE1060; 1:1000 for Western); RAD50 (Abcam #ab228935; 1:500 for Western); MRE11 (Abcam # ab30725; 1:1000 for Western); NBS1 (Abcam # ab23996; 1:2000 for Western); H2AX (Bethyl # A300-082A; 1:800 for Western); p-CHK2 (Cell Signaling #2661; 1:100 for IF); GST (Santa Cruz Tech # sc-138; 1:1000 for Western). Secondary antibodies used for Western (at 1:10, 000) are: donkey anti-rabbit IR Dye 800 (Licor Biosciences #926-32213); donkey anti-rabbit IR Dye 680 (Licor Biosciences # 926-68023); donkey anti-mouse IR Dye 800 (Licor Biosciences # 926-32213); donkey anti-mouse IR Dye 680 (Licor Biosciences # 926-68022); Streptavidin-conjugated IR Dye 800 (Licor Biosciences # 926-32230). Secondary antibodies used for IF (at 1:1000) are: goat anti-mouse Alexa Fluor 555 (Invitrogen # A21424); goat anti-rabbit Alexa Fluor 647 (Invitrogen # A22144); goat anti-rabbit Alexa Fluor 488 (Invitrogen # A11034).

Validation

All commercial antibodies have been validated by published studies (see below for PubMed PMID) or on the manufacturer's site (link provided below, where required). Custom site-specific anti-acetyl SAMHD1 K354 antibody has been validated in Figure 2.

IgG, Invitrogen 10500C, IP/ChIP, PMID 30017589
IgG, Sigma N103, IP, PMID 33875784
SAMDH1, Origene TA502024, WB/ IP/ChIP, PMID 28834754
Pan-Ac, CST 94415, WB, PMID 19608861
GFP, Santa Cruz Tech sc996, WB, PMID 35173176;28834754
GFP, Abcam ab290, WB/ChIP, PMID 34918745
SirT1, Abcam ab32441, WB/IP,PMID 34108453; 32034146
SirT2, ThermoFisher (custom made), WB/IP, PMID 33789098
SirT6, Abcam ab62768, WB/IP, PMID 27568560
SirT7, Abcam ab62748, WB/IP, PMID 30420520
Flag, CST 2368s, WB/IP, PMID 28474680
Flag, Santa Cruz Tech sc51590, WB/IP, PMID 35264593
a-tubulin, Sigma T6074, WB, PMID 35264593
GAPDH, Sigma 9545, WB, PMID 35896528
gH2AX, CST 9718, WB/IF, PMID 36184605;4772932
gH2AX, Millipore 05-636, IF, PMID 33789098
BrdU, BD Bio 3475880, IF, PMID 28834754
RPA32, Santa Cruz Tech sc14692, WB, PMID 28834754
pRPA32 s4/8, Bethyl A 700-009, WB, PMID 28834754
CtIP, Millipore MABE1060, WB, PMID 36075911
Rad50, Abcam ab228935, WB, validated for WB in humans by Abcam (https://www.abcam.com/rad50-antibody-n-terminal-ab228935.html)
MRE11, Abcam ab30725, WB, PMID 28834754
NBS1, Abcam ab23996; WB, PMID 30017584
H2AX, Bethyl A 300-022A, WB , PMID 28834754
p-CHK2, CST 2661, IF, PMID 22373579
GST, Santa Cruz Tech sc-138, WB/IP, PMID 34599178

Eukaryotic cell lines

Policy information about cell lines and Sex and Gender in Research

Cell line source(s)

HEK293T, HCT116, HeLa and U20S mammalian cell lines were purchased from American Type Culture Collection (ATCC, Manassas, VA). U20S-235 mCherry-LacI-Fok1 cell line were provided by Dr. Roger Greenberg and U20S-DR-GFP cell line were obtained from Dr. Jeremy Stark. AsiSI-ER-U2OS (DIvA-DSB inducible via AsiSI) cells were provided by Dr. Gaëlle Legube.

Authentication

HEK293T, HCT116, HeLa and U20S mammalian cell lines were authenticated by ATCC via STR testing; Authenticated U20S

Authentication	was used to make U2OS-235 (Shanbhag, N. M., Rafalska-Metcalf, I. U., Balane-Bolivar, C., Janicki, S. M. & Greenberg, R. A. ATM-dependent chromatin changes silence transcription in cis to DNA double-strand breaks. Cell 141, 970-981, doi:10.1016/j.cell.2010.04.038 2010), U2OS-DR-GFP (Pierce, A. J., Johnson, R. D., Thompson, L. H. & Jasin, M. XRCC3 promotes homology-directed repair of DNA damage in mammalian cells. Genes & development 13, 2633-2638 1999) and U2OS-AsiSI-ER (Aymard, F. et al. Transcriptionally active chromatin recruits homologous recombination at DNA double-strand breaks. Nat Struct Mol Biol 21, 366-374, doi:10.1038/nsmb.2796 2014).
Mycoplasma contamination	Cell lines were mycoplasma free.
Commonly misidentified lines (See ICLAC register)	No commonly misidentified lines used.

Flow Cytometry

Plots

Confirm that:

☒ The axis labels state the marker and fluorochrome used (e.g. CD4-FITC).

☒ The axis scales are clearly visible. Include numbers along axes only for bottom left plot of group (a 'group' is an analysis of identical markers).

☒ All plots are contour plots with outliers or pseudocolor plots.

☒ A numerical value for number of cells or percentage (with statistics) is provided.

Methodology

Sample preparation	U2OS DR-GFP cells expressing GFP (and RFP where applicable) were harvested, washed with PBS twice and resuspended in 200 ul PBS. Samples were subjected to flow cytometry.
Instrument	Aurora Cytek
Software	SpectroFlo software was used to conduct the flow cytometry experiments in Aurora Cytek and FlowJo software was subsequently used to analyze the data.
Cell population abundance	RFP population ranged between 40-60%, indicating transfection efficiency of the DR-GFP U2OS cells. Within the RFP population, GFP positive cells ranged from 1-8% (when there is homologous recombination present).
Gating strategy	Samples were first gated for % cells using FSC/SCC plots and then the cells were gated for RFP positive cells. Within the RFP positive cells, cells were gated for GFP population as an output for HR efficiency.

☒ Tick this box to confirm that a figure exemplifying the gating strategy is provided in the Supplementary Information.
